# A Cluster Randomized Controlled Trial of a Total Worker Health^®^ Intervention on Commercial Construction Sites

**DOI:** 10.3390/ijerph15112354

**Published:** 2018-10-25

**Authors:** Susan E. Peters, Michael P. Grant, Justin Rodgers, Justin Manjourides, Cassandra A. Okechukwu, Jack T. Dennerlein

**Affiliations:** 1 Harvard Center for Work, Health and Wellbeing, Harvard T.H. Chan School of Public Health, Boston, MA 02115, USA; sepeters@hsph.harvard.edu (S.E.P.); mgrant@hsph.harvard.edu (M.P.G.); cassandrao@post.harvard.edu (C.A.O.); 2 Department of Health Sciences, Bouvé College of Health Sciences, Northeastern University, Boston, MA 02115, USA; j.rodgers@northeastern.edu (J.R.); j.manjourides@northeastern.edu (J.M.); 3 Department of Physical Therapy, Movement and Rehabilitation Science, Bouvé College of Health Sciences, Northeastern University, Boston, MA 02115, USA

**Keywords:** organizational intervention, health promotion, injury prevention, musculoskeletal, ergonomics, mixed-methods study, construction industry, safety management, health risk behaviors, occupational health

## Abstract

This study evaluated the efficacy of an integrated Total Worker Health**^®^** program, “All the Right Moves”, designed to target the conditions of work and workers’ health behaviors through an ergonomics program combined with a worksite-based health promotion Health Week intervention. A matched-pair cluster randomized controlled trial was conducted on ten worksites (five intervention (*n* = 324); five control sites (*n* = 283)). Worker surveys were collected at all sites pre- and post- exposure at one- and six-months. Linear and logistic regression models evaluated the effect of the intervention on pain and injury, dietary and physical activity behaviors, smoking, ergonomic practices, and work limitations. Worker focus groups and manager interviews supplemented the evaluation. After controlling for matched intervention and control pairs as well as covariates, at one-month following the ergonomics program we observed a significant improvement in ergonomic practices (B = 0.20, *p* = 0.002), and a reduction in incidences of pain and injury (OR = 0.58, *p* = 0.012) in the intervention group. At six months, we observed differences in favor of the intervention group for a reduction in physically demanding work (B = −0.25, *p* = 0.008), increased recreational physical activity (B = 35.2, *p* = 0.026) and higher consumption of fruits and vegetables (B = 0.87, *p* = 0.008). Process evaluation revealed barriers to intervention implementation fidelity and uptake, including a fissured multiemployer worksite, the itinerant nature of workers, competing production pressures, management support, and inclement weather. The All the Right Moves program had a positive impact at the individual level on the worksites with the program. For the longer term, the multi-organizational structure in the construction work environment needs to be considered to facilitate more upstream, long-term changes.

## 1. Introduction

Internationally, construction workers have higher rates of musculoskeletal disorders, and chronic diseases related to obesity, lack of physical activity and smoking than workers in other industries [1,2,3,4]. In construction workers, musculoskeletal disorders have a one-year pain prevalence rate (at least one episode of pain in the last year) of 51% for the back, 37% for the lower extremities, 32% for the upper extremities, and 24% for the neck in construction workers [5]. The high prevalence of musculoskeletal and cardiovascular disorders causes a sizable burden to employers, insurers, and society as a whole, attributing to work absenteeism, healthcare costs, work schedule delays, and high turnover [6,7,8]. In 2014, approximately 33% of absenteeism was attributed to musculoskeletal symptoms [9].

Construction workers also have high rates of chronic health issues. Over 70% of construction workers are overweight [10]. Obese construction workers are at increased risk of receiving disability benefits for cardiovascular disease and musculoskeletal disorders [11,12]. This risk is even higher for obese workers with high physical job demands, especially for those with musculoskeletal disorders [11]. Specifically, construction workers also have the highest prevalence of smoking (39%) of all occupational groups [1]. The risk of chronic lung disease and cancers is also amplified by the combined effects of smoking with other respiratory exposures, such as dust, silica, and asbestos [13,14,15].

Extensive research has linked these injury and poor health outcomes to individual factors, as well as the conditions of work, including job demands, physical work environment and psychosocial work factors (e.g., supervisor support and worker collegiality) [7]. Construction workers’ injuries and poor health have been associated with the high physical demands, prolonged exposure to awkward postures, whole body vibration, long working hours, and psychosocial hazards in the work environment [2,16].

While these factors are prevalent in the construction industry, the complex work organization of construction work provides additional challenges for implementing traditional workplace prevention programs. The hierarchical structure between the site owners, general contractors, and subcontracting companies results in a fissured workplace [17]. The dynamic nature of these worksites results in workers moving on and off the site day-to-day. In addition, construction has high workforce turnover within the company, further complicating the number of workers transitioning in and out of the workforce, which has been linked to higher injury rates [18,19].

Integrated approaches that address the work environment to improve both occupational safety and health outcomes, and worker wellbeing outcomes, are acknowledged as being the most successful [20,21]. However, these integrated and comprehensive interventions for construction worksites need further investigation [20,21,22,23,24,25]. To date, most integrated approaches for construction workers have been individual-based, or those provided through labor unions [24,26]. Many worksite-based safety interventions for construction worksites have focused on using simple campaigns (such as poster and leaflet educational material) [27,28], training programs [29,30], behavioral management programs [10,31,32], or new, task-specific ergonomic tools and methods [33,34]. The intervention, “All the Right Moves” (ARM) described in this study, tested a different approach based on integrated approaches promoted by The National Institute for Occupational Safety and Health (NIOSH) Total Worker Health*^®^* program. Such approaches target the conditions of work that affect workers injuries and health outcomes [7,35]. The ARM intervention targeted the conditions of work through a worksite-based ergonomics program integrated into current work practices and on-site opportunities for workers to improve their health behaviors. The project’s goal was to develop and determine the feasibility of an integrated health promotion and health protection worksite-based program designed specifically for the dynamic nature of a commercial construction work site.

The purpose of this study was to examine the intervention—ARM, on commercial construction sites, using a mixed methods approach. The specific aims of this project were to examine the efficacy of an integrated program including: (1) a soft tissue injury prevention program on workers’ perception of worksite ergonomic practices, new pain and injury incidences, and work limitations; and (2) a health promotion/health coaching (Health Week) program for diet, leisure time physical activity, and reduced smoking behaviors.

## 2. Materials and Methods

### 2.1. Study Design and Randomization

We conducted a cluster randomized control trial on ten (five matched pairs) commercial construction sites (five intervention; five control) across the Boston metropolitan area, Massachusetts, United States between 2014 and 2015. Construction sites were matched within each general contracting company that agreed to participate in the study. Each pair of worksites was matched based on approximate size, scope, and phase of construction. This ensured that each matched pair consisted of similar organizational and worksite factors, such as similar existing company health and safety management systems [36]. Within each matched pair, one site was randomly assigned to either an intervention or a control group. A blocked randomization sequence was generated using a web-based random number generator by a member of the research team, who then allocated the pairs to either intervention or control. All workers within a specific worksite received the same intervention (or control) as allocated. Randomization and allocation of randomization sequence occurred as soon as two construction sites within a general contractor agreed to participate regardless of their assignment to control or intervention. The intervention groups received the intervention, ARM, whilst the control group received no intervention. Due to the pragmatic nature of the intervention, neither interventionists nor participants were blinded.

### 2.2. Recruitment and Eligibility

Construction sites were recruited through construction site owners and general contractors. To be eligible to participate in the study, worksites had to be in operation for 4 months or longer, and have 30 or more workers. Before the intervention commenced, a recruitment meeting was conducted with each site owner or general contractor to provide an overview of the study, programmatic activities, and to obtain leadership commitment. These recruitment meetings were conducted by the intervention primary investigator (J.T.D). Once a general contractor agreed, additional meetings with the leadership of each of the selected sites provided further leadership commitment and agreement for the study to take place on their sites.

Workers were introduced to the study by the research team and the general contractor safety manager, at a “safety stand-down” or toolbox talk. During the study, all new workers on a site were oriented to the study at their new-worker onsite safety orientation. Individual construction workers were surveyed within each site after a study launch meeting and at new-hire safety orientations for those workers who started after study commencement. Surveyed workers at each site were eligible if they were aged 18–65, and were English literate. All surveyed workers self-nominated and provided verbal consent during the survey process

All subjects gave their informed consent for inclusion before they participated in any data collection activities. The study was conducted in accordance with the Declaration of Helsinki, and the protocol was approved by the Ethics Committee of Harvard T.H. Chan School of Public Health Institutional Review Board (IRB-13-1948).

### 2.3. The “All the Right Moves” (ARM) Intervention

The ARM intervention was designed to integrate intervention components into the companies’ existing safety and health practices on the sites. The intervention components were first developed and vetted with researchers and construction safety professionals, based on evidence-based organizational interventions and our own studies in the construction industry [4,18,37]. Following this process, the components of the interventions were piloted on commercial construction sites not involved in this trial. Managers and workers from the pilot sites provided qualitative feedback on program components, and the feedback was used to modify the intervention components. The changes were again vetted with these workers to refine the intervention components. This feedback was crucial to ensure intervention-organization fit, worker buy-in, and feasibility of implementing the components.

**Figure 1:**
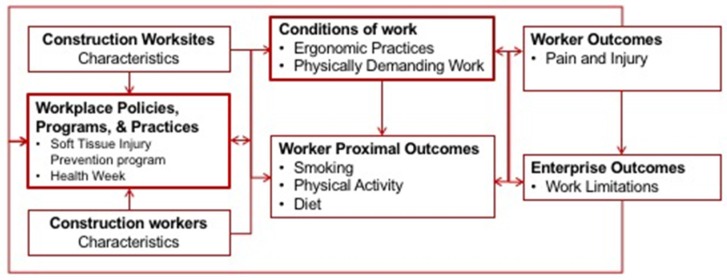
Logic model for the All the Right Moves (ARM) intervention.

The ARM intervention contained two main intervention components: (1) the Soft Tissue Injury Prevention Program (StIPP) which focused on improving ergonomics practices at the site and worker level to improve musculoskeletal health; and (2) Health Week, that integrated key messages and provided integrated health coaching opportunities for individual workers to improve ergonomic practices and improved health behaviors (diet, physical activity, and smoking) associated with cardiovascular health. Both of these activities were based on industry safety practices with the ergonomics program using a structure similar to current safety management systems [38], and the health week based on the industry’s practice of Safety Week campaigns and training. Refer to Figure 1 for the intervention’s logic model.

*The StIPP intervention component:* This consisted of worksite inspections and feedback, task pre-planning, supervisor training, and worker training implemented for six weeks prior to the health week. The ergonomics-focused program targeted organizational practices and physical job demands by creating a systematic process to control worksite hazards.

Worksite Inspections and Feedback: The inspection process utilized a standardized worksite walkthrough inspection process augmented from an existing safety inspection process adapted from the successful Building Safety for Everyone program [18,36]. Photographs were taken of the injury hazards and ergonomic solutions which could be uploaded through an internet-based platform. The internet-based platform allowed data from all observations in a given date range to be aggregated and a report generated. A pre-intervention worksite inspection was conducted for each site one-week before the intervention activities were launched in order to customize foreman training as well as provide one-on-one training for the safety manager to identify soft tissue injury hazards and ergonomic practices. The walkthrough was conducted by an experienced ergonomist (J.T.D or M.P.G.), who was accompanied by a research assistant and the site safety manager from the general contractor. During the following six-week intervention periods, the safety manager conducted safety inspections on their own documenting their inspections using a custom-made web-based inspection tool. The tool allowed the safety manager to upload observations, including date, location, photo, hazard identified, solutions.

Each week the research team working with the safety manager compiled an inspection report and materials to provide critical feedback to the foreman and to the work crews. Based on our learnings from the Building Safety for Everyone program [18], detailed reports were communicated to foreman at weekly meetings and posters highlighting examples of hazards and solutions from these reports were placed in highly-visible areas around the worksite.

Task pre-planning: In addition to the inspection and feedback, we adapted existing pre-task planning checklists to incorporate soft-tissue injury hazards and the application of ergonomics solutions. These checklists identified task that involved manual materials handling, overhead work, and ground work. Ergonomic solutions included the NIOSH Simple Ergonomics Solutions for Construction Workers [39], as well as various trade specific solutions publicly available, which we compiled in a manual for the safety managers.

Supervisor training: This took place at the start of the intervention to report information from the pre-intervention walkthrough to the site foreman for the subcontractor companies currently on the site. The training curriculum included information about the intervention, programmatic activities, injury hazards and ergonomic solutions identified from the first worksite inspection, and a few basic solutions from the NIOSH Simple Solutions [39], as well as expectations for the duration of the intervention implementation. The training was conducted during a mandatory weekly foreman meeting. Safety managers for each site also received this training, as well as trainings on how to use the web-based worksite inspection tool. 

Worker training consisted of an “Ergonomics Toolbox Talk” (i.e., full company break in normal work to discuss an observed safety concern) that consisted of providing a few of the key messages from the supervisor training. The toolbox talk took place at the start of the intervention for workers already on the worksite and during new worker safety orientations, for workers coming onto the site after the initial launch meetings.

Health Week: This health promotion intervention was modeled after the existing safety week in construction (one week each year that is dedicated to raising awareness of workplace safety). The key goal of Health Week was to provide health education through toolbox talks and engage workers in programs to facilitate health behaviors through an opt-in health coaching program. Health Week targeted psychosocial factors and individual health-related behaviors by engaging workers in one-on-one discussions about their health and connecting them with relevant resources to improve their health behaviors. Toolbox talks were held during workers’ break times each day of Health Week. Scripts and one-page toolbox cards were developed by the research team and a health promotion consultant, and then vetted with construction companies before being used. Topics included benefits of health coaching, soft tissue injury prevention, smoking cessation, energy balance (diet and physical activity) and a wrap-up session. Free web-based and phone resources were provided for each relevant topic. In addition, resources included free telephone-based health coaching provided by a large health-care organization. For active smokers, nicotine replacement therapy (NRT) (two-week supply) was provided free of charge.

Because of the success of individualized health coaching in construction workers, and prior results demonstrating dynamic workers movement between sites, Health Week encouraged workers to sign up and participate in health coaching program [40,41]. The health coaching program consisted of up to four telephone sessions by a trained health coach at no cost to the worker. The focus of these sessions was soft tissue injury prevention, dietary behaviors, physical activity and smoking cessation. Workers were able to select which of these topics they would receive coaching for. Each day workers were reminded to sign up for health coaching and those who did sign up were put into a lottery to win a USD$50 gift card to a large hardware-chain store.

### 2.4. Control Site Activities

For the control sites, all workers completed surveys at the same time intervals as the intervention sites. Workers were introduced to the study at an initial toolbox talk or at new worker orientation. They were also asked to complete surveys, at the same time intervals as the intervention sites. For the data collection periods, a banner with the program’s logo was posted on the control group sites, similar to the intervention sites. No other information was provided, and no other activities were completed on control sites.

### 2.5. Worker Survey Data Collection

Workers completed surveys on-site at baseline—either at the initial work-site toolbox talk or at new worker orientations for workers joining the site after the launch of the project. Workers also completed surveys at two follow-up intervals, after completion of the StIPP program and one week prior to health week (FU1), and six months after health week (FU2). Due to the flow of workers on site, follow-up 1 occurred from 1 to 5 weeks post baseline survey. Baseline and FU1 surveys were collected on site and FU2 was collected via mail delivery. Workers were incentivized with a USD$5 gift card for completion of FU1 surveys, and USD$20 for FU2 surveys. The surveys contained questions on perceived work environment, ergonomic practices, health behaviors and worker health outcomes. The baseline survey also contained additional sociodemographic questions. The primary outcomes were health outcomes (pain and work limitations). Secondary worker proximal outcomes included health behaviors and safety practices. Workers who consented to participate were tracked via phone call or text to complete follow-up surveys at one (FU1) and six months (FU2) if they were no longer at the original study site.

### 2.6. Primary and Secondary Outcome Measures

Musculoskeletal pain and injury was measured with items adapted from the Nordic MSK Pain instrument [42]. We examined the probability of a worker having a new incidence of pain and/or injury at the follow-up time points using the question: “Have you needed to reduce or alter your work because of injury or musculoskeletal pain?” The questionnaire timeframe was adapted for the three different surveys to allow us to capture a change in pain and/or injury incidence following intervention roll-out. For baseline, the respondents were asked to answer the question with respect to last 12 months. For FU1, this was since they started work on the control/intervention site, and for FU2, it was for the preceding 6 months. Pain (without injury) was measured by asking: “During the past 3 months, have you had pain or aching in any of the areas shown on the diagram?”

Dietary behaviors were measured with three variables: “healthy diet”, “unhealthy diet”, and “dietary balance” at FU2 only [43,44]. Healthy diet was measured using six questions about the weekly frequency with which participants consumed the following types of foods and beverages: fruits, 100% orange or grapefruit juice, other 100% fruit juices, vegetables, baked potatoes, and salad. Unhealthy diet was measured with questions on the weekly consumption of fried foods, sugared snacks, fast foods, and sugar-sweetened beverages. Dietary balance was calculated as the sum of healthy diet and unhealthy diet multiplied by negative one, so that positive dietary balance indicated a healthier diet and negative dietary balance indicated a less healthy diet.

Physical activity was measured using a modified version of the Centers for Disease Control and Prevention Behavioral Risk Factor and Surveillance System Physical Activity Measure at FU2 only [45]. It included items on time spent walking and participating in both vigorous and moderate physical activities both at home and work during the last seven days.

Smoking status was categorized as a current smoker, former smoker, or never smoker [46]. Current smokers were those who currently smoke and have smoked at least 100 cigarettes in their lifetime. Former smokers were those who had smoked at least 100 cigarettes in their lifetime but do not currently smoke. Never smokers were those who had smoked less than 100 cigarettes and do not currently smoke. Current smokers were further differentiated according to the magnitude of their smoking behavior, measured by smoking frequency, smoking quantity, and contemplation.

### 2.7. Other Variables Measured

Ergonomic Practices were measured using three items from Amick et al. [47]: “Ergonomic strategies are used to improve the design of work”, “Ergonomic factors are considered in task pre-planning and in purchasing new tools or equipment”, and “Ergonomic factors are considered in safety and health inspections”. These were rated on a 5-point scale ranging from 1 = strongly agree to 5 = strongly disagree. Ergonomic practice items were coded for the analysis so that higher scores of ergonomic practices represented better ergonomic practices.

Work limitations were measured using the eight question short-form Work Limitations Questionnaire, which contained domains on time, physical, mental and interpersonal demands [48]. Responses on a 5-point Likert scale were coded for the analysis so that higher values on the work limitations scale represented more or higher work limitations.

Physically demanding job demands were measured by two ordinal variables stemming from the following questions: “Please indicate how physically demanding your job is over the last 7 days”, on a scale ranging from 1 = “not at all physically demanding” to 5 = “extremely physically demanding”.

Sociodemographic variables included age (years), sex (male/female), race (white, black/African American, Latino/Hispanic, other), education level, job title (apprentice, journeyman, foreman and supervisor), and construction trade (carpenters, electricians, drywallers, ironworkers, laborers, painters, pipefitters, and plumbers). Race and ethnicity were later combined into the following two categories: “white” and “not white” for the analysis. Job title was later categorized into two categories for the analysis: “apprentice/journeyman”, and “foreman/supervisor”. Trade was categorized into the following four categories based on workers’ job demands: (1) mechanical; (2) finishing; (3) ironwork; and, (4) labor. These categorizations have been used previously [36].

### 2.8. Process Evaluation

Process evaluation data collection focused on collecting information on uptake and exposure to the intervention components, as well as barriers and facilitators to implementation. Qualitative data was collected at the completion of the intervention through focus groups with workers and interviews with managers. All interviews and focus groups were recorded and transcribed. To maintain confidentiality, participants were instructed to avoid identifying themselves, their coworkers or the company they worked for, during the interviews and focus groups. In addition, data were collected on uptake and exposure to the intervention components through checklists completed for each intervention activity by members of the research team.

Post-intervention worker focus groups were conducted at four of the five intervention sites. One site did not participate due to a scheduling conflict. The aims of the focus groups were to: (1) explore workers perceptions of health and safety at their sites; (2) explore workers’ perceptions of intervention activities including facilitators and barriers to uptake of the intervention, feasibility, and success of the intervention; and (3) identify how health and safety was handled onsite considering the fissured nature of the worksites and workforce.

Post-intervention interviews with safety managers from the general contractors were conducted at the same seven worksites in which worker focus groups were conducted. The aim was to: (1) explore their perceptions of intervention activities including facilitators and barriers to uptake of the intervention and intervention delivery, feasibility, and success of the intervention; (2) investigate mechanisms that enable foremen and site management to support worker participation in health and safety interventions; and, (3) identify areas for improvement for future interventions.

### 2.9. Hypotheses

We tested the following hypotheses for the primary outcomes: (1a) At FU1 and FU2, workers on intervention sites will report lower incidences of pain or injury compared to workers on the control sites; (1b) At FU2, workers on intervention sites will report improved diet and leisure time physical activity behaviors compared to workers on the control sites; and (1c) At FU2, workers on intervention sites will smoke on fewer days and with fewer cigarettes per month.

We also tested hypotheses for secondary outcomes: (2a) Workers on intervention sites will report improved ergonomic and safety practices at FU1, and lower physical job demands at FU1 and FU2, compared to workers on control sites at follow up; and (2b) At FU1 and FU2, workers on intervention sites will report improved work limitations at follow up than workers on the control sites.

### 2.10. Data Analysis

All data analyses were completed in SAS Version 9.3 (SAS Institute Inc., Cary, NC, USA). First, we compared worker demographics between control and intervention sites using chi-squared tests of homogeneity for categorical variables and *t*-tests for continuous variables. A priori power calculations were conducted for the primary outcome, pain and injury, adjusting for potential intra-class correlation, ICC = 0.05 due to the cluster-based design, and using a two-sided test at α = 0.05. We have sufficient power (>0.8) to detect at effect size greater than 0.6 with an estimated sample size of 176.

As pain and injury outcomes were binary measures, we first performed logistic regression models accounting only for the baseline level of the outcome variables. Each model also utilized cluster robust standard errors to account for individual correlation within worksites. Second, we included fixed effects for the matched pairs within each company, and adjusted the models for age, race, and job title.

All other variables were continuous. We conducted linear regression models on the change scores between baseline and FU1 and, baseline and FU2 as the dependent variables and treatment status (intervention; control) as the independent variable. We used cluster robust standard errors to account for individual clustering within worksites. We then adjusted for matched pairs within the companies through the addition of a fixed effect and also accounted for the possibility of post-randomization, and residual confounding by adjusting for age, sex, race, job title, and trade. No analyses were conducted for smoking as there were too few smokers who changed their smoking status over the course of the intervention on the sites.

We conducted sensitivity analyses to observe initially whether the removal of the one matched pair for the site that did not perform the intervention activities for the soft tissue ergonomics program, resulted in any differences in effect of the intervention on the primary and secondary outcomes. We then sequentially removed each pair per analysis to evaluate whether removal of any pair resulted in differences in the effectiveness evaluation.

## 3. Results

### 3.1. Study Characteristics and Response Rates

**Figure 2:**
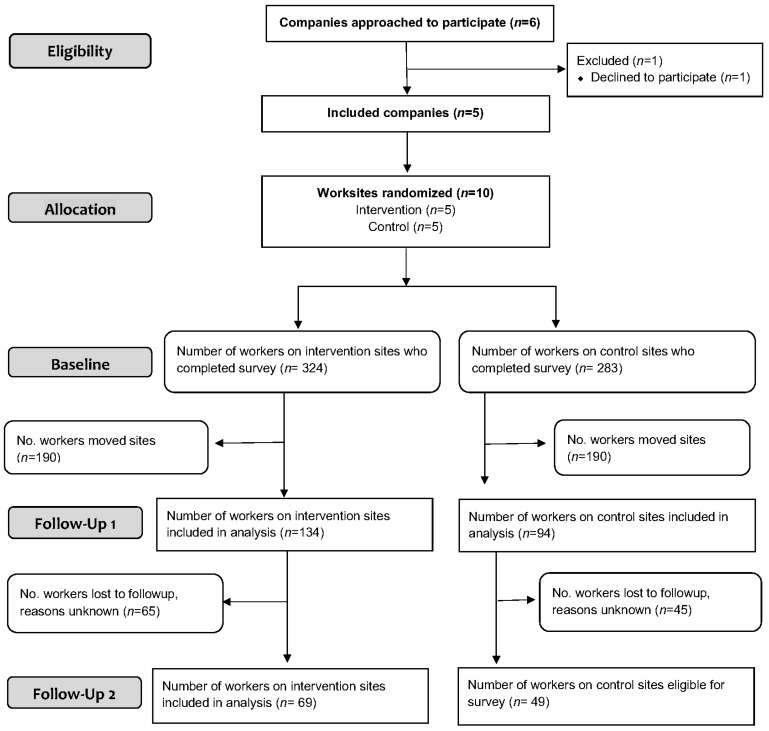
Participant flow through the trial.

Six construction companies operating in the Boston metropolitan area, Massachusetts, were invited to participate, and five agreed. Within each of these companies two worksites per company were randomly assigned to the intervention or the control, resulting in a total of 10 worksites. The participation rates for the follow-up surveys (of those who completed the baseline surveys) were 69% (*n* = 228/332) for FU1 and 78% (*n* = 118/151) for FU2, and were included in the analyses (Figure 2). At baseline, it was difficult to determine the total number of eligible workers on site and hence response rates of those eligible. We were able to record the number of workers at the site orientations and were able to capture almost all of the new workers that came on to the site after the orientation.

There were no significant differences between the intervention and control sites at baseline (Table 1), FU1 or FU2 for age, sex, race/ethnicity or education at any data collection interval. There were no significant differences (*p ≥* 0.05) for those who completed surveys at baseline and those who did not at FU1 or FU2, with respect to age, sex, ethnicity/race, education level or job title.

### 3.2. Outcomes

Similar to demographic characteristics, all the outcome variables were not statistically different between the workers in the intervention and control groups at baseline, except for physical demanding work (*p* < 0.001) (Table 2). However, as described in Section 2.10, models testing the hypotheses examined changes from baseline in the cohort accounting for baseline measurements.

#### 3.2.1. Pain and Injury

Hypothesis 1a tested differences in pain incidence between intervention and control group workers. Model results revealed no significant differences at FU1 in the unadjusted model. However, after adjusting for covariates, in addition to the matched pairs, there was approximately 42% reduction in risk of having new pain or injury compared to the control sites (*p* = 0.012) (Table 3). While the magnitude of this risk reduction was maintained at FU2 there were fewer participants and an increase in variability that made this reduction not statistically significant. Thus, hypothesis 1a was partially supported at FU1.

#### 3.2.2. Physical Activity and Dietary Behaviors

The number of minutes that participants spent performing recreational physical activity decreased on average in the control groups, but increased in the intervention groups. This difference was non-significant in the unadjusted model, but became significant in the adjusted model (B = 31.03, *p* = 0.03) (Table 4).

There were no observable differences at FU2 between the intervention and control sites for unhealthy diet (i.e., eating fatty or sugary foods) for the unadjusted or adjusted models respectively (Table 4). For healthy diet, we observed no differences between the intervention and control groups in the unadjusted model. However, when accounting for the matched intervention and control pairs within each company, and adjusting for covariates, we found a significant small positive influence on healthier diet behaviors in the intervention compared to the control groups (B = 0.87; *p* = 0.008). Overall, we saw a small improvement in having a more balanced diet nearing significance due to the improvement in healthy eating behaviors (B = 1.05, *p* = 0.054). Thus, hypothesis 1b was partially supported.

#### 3.2.3. Tobacco Use

Changes in smoking and tobacco used were small in both groups. Two people in the intervention group quit smoking, while in the control group, one person quit and one started smoking.

#### 3.2.4. Ergonomic Practices and Work Limitations

After the StIPP intervention activities, we observed a small but significant improvement in the intervention, compared to the control sites, for ergonomic practices, after adjusting for matched pairs, and age, gender, race, job title and trade (B = 0.20, *p* = 0.002) (Table 5). We also saw a significant small reduction in physical job demands at FU2 (B= −0.25, *p* = 0.008). Hypothesis 2a was therefore partially supported. There were no observable differences between the intervention and control sites in the worker’s perceptions of their work limitations at FU1 or FU2 for the unadjusted or adjusted models (Table 5). Thus, hypothesis 2b was not supported.

#### 3.2.5. Sensitivity Analysis

We conducted analyses by removing each matched pair across intervention and control sites. We observed that when we removed the matched pair which included the intervention site that had limited participation in the ergonomic intervention activities, the strength of the significant findings increased.

### 3.3. Process Evaluation

#### 3.3.1. Intervention Fidelity and Uptake

Soft Tissue Injury Prevention Program: Foreman training ranged from 25–45 min per site, and was delivered as per the protocol on the five intervention sites. The number of foreman who attended per site varied (median: 6; range: 5–25). Baseline participation rates for the project launch (range: 25–93%) and orientation (range: 75–90%) also varied significantly across sites. The number of ergonomic inspections and feedback reports differed greatly across the five intervention sites (median: 15; range: 0–19). At best, sites had three ergonomic observations per week during the six weeks of the program. At worst, one site completed no inspections and feedback reports to the foreman, due to severe weather conditions causing the site to shut down during the intervention period. Many of the improvements recorded concentrated on how workers were setting up their own work areas rather than systems level changes, e.g., getting equipment off the ground and performing tasks at heights around waist level, rather than below the knee.

Health Week and Coaching: 45 workers (14%) signed up for health coaching. Most workers had favorable responses to engaging in the toolbox talks during health week. However, only 7 out of the 45 workers who signed up for health coaching participated in the first phone call, and only three completed four weeks of health coaching.

Workers signed up for health coaching for dietary behaviors, physical activity and smoking cessation. No workers signed up for coaching on soft tissue injury prevention. Qualitatively, workers reported getting benefit from the smoking cessation toolbox talks. Providing NRT kits was popular; however, due to privacy issues and poor follow-up rates, we were unable to link the NRT distribution to the surveys and effects on smoking quit rates. One worker reported: “I think tobacco was good for me and my guys. Most of them smoke, so I think it was good for them. The NRT inspired some of the workers to give quitting a try.” Other topics of interest raised in the focus groups included stress management, alcohol consumption and appropriate pain management.

There were no adverse events reported by the participants for participating in either StIPP or Health Week.

#### 3.3.2. Barriers to Intervention Implementation

Based on key informant interviews on the intervention sites, while indicating it was good to have the ergonomics inspections and topics at the forefront of the workers’ and subcontractors’ activities, workers mentioned a number of barriers to fully implementing the intervention. 

Fissured workplace issues: A key barrier was the capability of subcontractor companies to make changes in working conditions. While the program trained the foreman of the subcontractors with a focus on pre-task planning, the subcontractors did not have the systems in place or the available tools to assist in changing the working conditions.

Production pressures and unpredictable schedules: The site that conducted no ergonomics inspections had large production pressures as the construction schedule was delayed significantly due to unusual winter weather. For example, one safety manager observed that production pressures could be a driving factor: “I think it’s the schedules… Because they rush around, it’s hard for them to take a step back and really evaluate how they’re doing things. They’re just trying to do it as quickly as possible.”

Management support and worker buy-in: Focus group participants and key informants reported that programs needed buy-in and support from upper management for interventions to be successful. This is especially true with respect to training and data collection which, by necessity, must be conducted on the worksite during working hours. For instance, general contractors could allow for extra training related to the ARM program and build it into the contracts of the subcontractors. That way, the time needed for training purposes and intervention delivery would be agreed to ahead of time and budgeted into the contracts signed by both parties. To illustrate this point, one safety manager noted: “A health and safety program would have a lot more buy-in and success on a site if it was written into the contract... An owner or GC [general contractor] would have to financially support the program running on their site.”

## 4. Discussion

The goal of this study was to evaluate the efficacy of a construction worksite-based integrated intervention targeting both the conditions of work, and workers’ health behaviors, simultaneously. We observed short-term improvements in ergonomic practices and in incidences of pain and injury after an injury prevention program. We also observed an improvement in physical activity and healthier dietary behaviors, such as increased consumption of fruits and vegetables, after a health promotion Health Week program.

At the individual level, we found a significant improvement in ergonomic practices, and a reduction in incidences of pain and injury, which supported the hypothesized pathways for the program. As promoted by NIOSH, ergonomic practices focus on workers modifying or establishing work procedures to reduce the risk of injuries [39,49]. While we did not quantify exposure to specific ergonomic hazards, the StIPP focused on workers’ setting up their work more ergonomically. For example, working at knuckle level instead of on the ground, and using appropriate tools to reduce extreme postures associated with overhead work and manual materials handling. The program targeted the conditions of work directly controlled by the workers themselves (Figure 2) [7]. Giving such control to workers is important in reducing disability, as it gives workers opportunities to adapt their work in order to better manage their own musculoskeletal symptoms and health [50,51].

While we were encouraged that an improvement in ergonomics practices occurred, results also indicated that the program was not successful at addressing system level components. For example, while we saw ergonomics practices improve, we saw no significant change in the physical demands on the workers. Hence, we suspect that the intervention changed the way people completed their work but had limited effect on the physical demands of the job. In addition, the process evaluation revealed several important barriers and facilitators to our program at the organizational level. First, management and worker buy-in were identified to be integral to the success of the soft tissue injury prevention program. This was perceived to be key in a work environment that is fast-paced, unpredictable, and with tight production schedules tied to the requirements of the general contractor. For example, there was little time to complete task preplanning or for the safety manager to complete inspection protocols for injury hazards and ergonomic solutions. When management support for health and safety programs is not observed by the workers, other competing factors are often prioritized over health and safety, especially ergonomic practices [52]. This was quite evident on one site which had major delays due to the winter storms of 2015 in the Boston metropolitan area. Due to the loss of almost a month of production, competing safety and schedule priorities would supersede program delivery. Similar challenges to program delivery have been reported by others in the construction industry [53].

While we have had past success with a worksite safety program integrated within the complex structure associated with multiple employers, a large barrier to a systems approach ergonomics program was the challenge faced by subcontractors to make changes, even those changes that could improve site safety on their own worksite. Unlike our previous program that was designed to re-enforce existing safety practices [36], the ARM program required subcontractors to implement new, or modify existing practices and tools, that may be specific to their trade. Our program focused on simple ergonomics solutions that individual workers could implement to their own work [39]. However, more complex or system-level changes would require the involvement of multiple groups or stakeholders [54]. Ergonomics solutions in a fissured workplace require all site employers to take on elements of the program to effectively and systematically influence the overall conditions of work [17].

Moreover, system-level changes require better upfront planning before construction begins, such as during the bidding process for a job by setting out requirements from the multiple employers, and in the contracts for the jobs. The key informant interviews supported this concept. Expectations regarding safety programs in the contract is standard procedure in larger projects, especially owner-insured programs. An example is with respect to safety training, in which owners, especially public entities, require that construction workers have a minimum of OSHA-10 training to be onsite [55]. Whilst others require their contracting companies complete safety prequalification safety surveys, or have written safety management programs. Thus, including ergonomics in the contractual language may set up better expectations for a program.

Other researchers in the construction industry have also found mixed findings with respect to improvements in pain and injury and perceived physical effort after implementing ergonomic interventions, including participatory ergonomics programs [33,56,57,58]. In these studies, reasons for intervention failure were generally associated with the intervention not being delivered as intended or implemented at all of the sites [28,57]. In our study, intervention delivery occurred as per the protocol in four of the five sites during the intervention period. However, since the ergonomics program stopped after six weeks and workers often moved from sites before the follow-up data collection was completed, we also attributed this to our loss of significance at the six-month follow-up. Although we observed that on average the reduction in pain and injury incidences, and improvements in ergonomic practices were maintained, there was reduction in power due to loss to follow up.

In contrast, the Health Week had many successes in overcoming some of these barriers associated with the multiemployer structure. For one, it simply required the participation of the workers and little, if any, infrastructure. In theory, the Health Week might have addressed some conditions of work regarding psychosocial factors around health, like supervisor or co-worker support. Anecdotally, we observed foremen and co-workers being supportive of ensuring their co-workers signed up for health coaching or NRT. Some foremen would cover for their workers to allow them to participate in the week’s activities. We also observed workers talking about eating healthier food with their coworkers during the week.

Another major strength of the Health Week was how it aligned with companies’ current practices on the worksite and also with the interests and goals of the workers. This was also found in previous formative work we completed that found that policies, programs and practices are supported by management and workers alike if they can be easily integrated into company’s business structures and align with workers’ goals and needs [59,60]. Health Week was in a familiar format for the workers and companies alike. We modelled Health Week after the industry’s standard practice of safety week where contractors have a specific theme and perform a series of outreach activities for workers to provide information on resources and best practices. Thus, due to the familiar format, workers may have been more receptive to the daily topics. Although the uptake on NRT and individualized health coaching was low, we did see improvements in workers’ health behaviors, including higher intake of fruits and vegetables, and increased amount of time per week engaged in recreational physical activity. This finding is similar to the results of a health promotion intervention conducted in the Netherlands, which found that onsite group coaching sessions resulted in changes in physical activity, and dietary behaviors, but did not improve musculoskeletal symptoms [61].

### Methodological Considerations

Research in construction has challenges with loss to follow up due to the dynamic nature of construction with workers coming and going on worksites as the construction job requires specific trades during the timeline of the study [18,36]. The issue of poor follow-up rates can lead to bias; however, usually towards the null [62]. This is predominately due to the itinerant nature of construction workers [18]. This resulted in our analyses at FU2 being underpowered for some of our outcomes (such as pain and injury) where the effect size was similar to FU1. Similar findings have been found in related interventions [63].

Another challenge was the success of integrating the injury prevention and health promotion activities in this environment. Integration was achieved by linking the two programs by name and key messaging in the planning and implementation phases. In addition, messaging around Health Week included training on both injury prevention and health promotion giving workers the tools to improve their working conditions, as well as giving them control for their health. The ergonomics program prior to Health Week did have health messaging but without any specific health promotion activities.

One limitation was that our intervention depended on the participation of the general contractor safety managers, whose involvement and dedication to the study varied across sites and between general contractors. This aspect of the intervention was by design, as we considered it important for integration and sustainability into current company processes, that the ergonomics inspections were performed by the safety managers. Giving the safety managers latitude to decide how invested they were in the program allowed us to assess the feasibility of the intervention being adopted without the aid of study staff. This would ensure that our observations were realistic and representative of barriers and facilitators to the intervention’s delivery by non-study staff.

Further, this study involved worksites in commercial construction only. Thus, the results may not be generalizable to other types of construction (i.e., residential or industrial). However, commercial construction accounts for a large portion of U.S. construction activities, and represents an important area for injury prevention research. Similarly, the construction workforce in the Boston metropolitan area may not be representative of commercial construction workers in other parts of the country or world, where work practices, demographics, and union membership differ.

Despite these limitations, our study had several strengths, most notably the study design and the wide variety of general contractors and sites recruited into the study. The cluster randomized control trial design is a novel approach in commercial construction. Typically, approaches to improve the health and safety of construction workers have often focused on the individual worker, targeting workers when they are enrolled in apprentice programs [64,65], targeting workers through social media campaigns via posters at worksites and/or brochures sent to union members [66,67], and engineering controls for specific tasks [68]. However, best practice involves system-level approaches that comprehensively address workplace systems relevant to the control of hazards and worker safety, health, and well-being [20]. This study was fortunate to be able to recruit five major general contractors operating in the Boston metropolitan area and gain access to ten different construction sites for the purpose of evaluating the ARM intervention. Furthermore, delivering the intervention through mid-level managers (through a combination of the general contractor safety managers and subcontractor foremen) was a strength of the study. This focused intervention efforts on those who were in the best positions to make changes to the conditions of work.

The ergonomics inspection and communication protocol provided a method to identify broad areas for improving ergonomics in the dynamic construction work environment. It is important to understand the challenges and successes of intervention delivery in order to inform and improve future worksite-based interventions. It appears that the largest barriers to the success of the intervention were the inability of subcontractors to make changes to their worksite and the variability in the involvement and dedication to the study across different worksites and general contractors. These are real-world, as well as research study challenges. Subcontractors did not have the systems in place, or the available tools, to assist in changing their working conditions. Competing safety and production priorities also influenced the level of management commitment to the study. Additionally, construction safety research may have broader implications for an increasing number of industries that are becoming as dynamic and variable as construction, as more services once housed in a single facility are outsourced to multiple employers [17].

## 5. Conclusions

The ARM program had a positive impact at the individual level on the worksites that implemented the program. The trial saw improved ergonomics practices, as well as, reduction in new pain and injury, and improved diet and physical activity, as reported by the workers. A number of obstacles were encountered which made integrating a health promotion and injury prevention intervention into the multi-employer, outcome-driven, dynamic work environment challenging. Process tracking suggested that our intervention had less impact at the systems/organizational level in terms of changing organizational programs and practices, due to the complex organizational structures on site. For the longer term, more organizations in the multiple employer environment should be involved in the implementation to facilitate more upstream changes. 

## Figures and Tables

**Table 1:** Distribution of demographic characteristics at baseline (*N* = 607).

	Total (*N* = 607)		Control (*n* = 283)		Intervention (*n* = 324)		Test of Equivalence, *p*-Value
	*N*	Mean (SD)	*N*	Mean (SD)	*N*	Mean (SD)	
Age	586	40.42 (10.78)		40.28 (11.05)		40.55 (10.55)	0.7643
	***N***	***n* (%)**	***N***	***n* (%)**	***N***	***n* (%)**	
Gender	592		275		317		0.0736
Male		573 (97%)		270 (98%)		303 (96%)	
Female		19 (3%)		5 (2%)		14 (4%)	
Race/Ethnicity	595		244		351		0.7883
White		457 (77%)		214 (77%)		243 (77%)	
Black/AA		57 (10%)		24 (9%)		33 (11%)	
Latino/Hispanic		35 (6%)		16 (6%)		19 (6%)	
Other		46 (8%)		24 (9%)		22 (7%)	
Education	587		274		313		0.5762
<H.S.		33 (6%)		17 (6%)		16 (5%)	
H.S./G.E.D.		317 (54%)		151 (55%)		166 (53%)	
Some college		194 (33%)		90 (33%)		104 (33%)	
College graduate		43 (7%)		16 (6%)		27 (9%)	
Title	554		256		298		0.3400
Apprentice		104 (19%)		40 (16%)		64 (22%)	
Journeyman		330 (59%)		156 (61%)		174 (58%)	
Foreman		100 (18%)		50 (19%)		50 (17%)	
Supervisor		20 (4%)		10 (4%)		10 (3%)	
Trade	499		226		273		0.1643
Finishing		59 (12%)		25 (11%)		34 (12%)	
Mechanical		366 (73%)		168 (74%)		198 (73%)	
Laborers		30 (6%)		18 (8%)		12 (4%)	
Ironworkers		44 (9%)		15 (7%)		29 (11%)	

AA = African American, H.S. = High School, G.E.D. = General Equivalency Diploma; SD = Standard Deviation.

**Table 2:** Descriptive statistics for primary and secondary outcome variables.

Outcome Variable	Control			Treatment		
	Baseline (*N* = 283)	FU1 (*N* = 94)	FU2 (*N* = 49)	Baseline (*N* = 324)	FU1 (*N* = 134)	FU2 (*N* = 69)
Worker Outcomes						
	n (%)_[N]_	n (%)_[N]_	n (%)_[N]_	n (%)_[N]_	n (%)_[N]_	n (%)_[N]_
New pain or injury ^2^	83 (30.0%)_[277]_	20 (21.5%)_[93]_	15 (31.3%)_[48]_	115 (36.4%)_[316]_	27 (20.6%)_[131]_	15 (21.7%)_[69]_
Pain interfering with work	197 (69.9%)_[282]_	54 (57.5%)_[94]_	37 (75.5%)_[49]_	234 (72.4%)_[323]_	78 (58.2%)_[134]_	48 (69.6%)_[69]_
Current Smoker	82 (30.4%)_[269]_		12 (24.5%)_[49]_	99 (33.9%)_[292]_		18 (26.1%)_[69]_
	**Mean (SD)_[N]_**	**Mean (SD)_[N]_**	**Mean (SD)_[N]_**	**Mean (SD)_[N]_**	**Mean (SD)_[N]_**	**Mean (SD)_[N]_**
Physical activity	76.57 (76.70)_[229]_		67.69 (95.55)_[45]_	67.76 (61.95)_[270]_		69.04 (93.66)_[66]_
Healthy diet	5.60 (2.32)_[276]_		5.42 (1.67)_[48]_	5.62 (2.24)_[318]_		5.48 (2.01)_[69]_
Unhealthy diet	2.05 (1.21)_[274]_		2.12 (1.37)_[47]_	2.25 (1.31)_[317]_		1.76 (1.15)_[69]_
Diet balance	3.57 (2.48)_[274]_		3.24 (1.92)_[47]_	3.36 (2.46)_[317]_		3.72 (2.25)_[69]_
**Enterprise Outcomes**						
Work limitations	1.51 (0.64)_[277]_	1.39 (0.57)_[91]_	1.27 (0.41)_[49]_	1.53 (0.67)_[321]_	1.46 (0.62)_[128]_	1.27 (0.54)_[68]_
**Conditions of Work**						
	**n (%)_[N]_**	**n (%)_[N]_**	**n (%)_[N]_**	**n (%)_[N]_**	**n (%)_[N]_**	**n (%)_[N]_**
Demanding Work ^1,3^	213 (79.3%)_[269]_	43 (47.3%)_[91]_	30 (61.2%)_[49]_	206 (66.9%)_[308]_	60 (46.2%)_[130]_	31 (45.6%)_[68]_
	**Mean (SD)_[N]_**	**Mean (SD)_[N]_**	**Mean (SD)_[N]_**	**Mean (SD)_[N]_**	**Mean (SD)_[N]_**	**Mean (SD)_[N]_**
Ergonomic practices	3.89 (0.67)_[276]_	3.69 (0.78)_[93]_		3.8 (0.62)_[313]_	3.68 (0.80)_[132]_	

FU1 = Follow-up 1; FU2 = Follow-up 2; SD = Standard Deviation. ^1^ Differences between treatment and control groups at baseline, *p* < 0.05; ^2^ At baseline, this was measured as the number of workers who had pain or injury, at follow-up intervals this was measured as new pain or injury since baseline; ^3^ Physical demanding work was categorized as those nominating a 4 or 5 on the physically demanding scale.

**Table 3:** Effects of ARM intervention on Pain and Injury at 1-month and 6-months post-intervention while adjusting for baseline level of outcome variable.

Outcome Variable	Unadjusted			Adjusted ^1^		
	*N*	OR (95% CI)	*p*-Value	*N*	OR (95% CI)	*p*-Value
FU1 (1 month)						
New pain or injury ^2^	216	1.01 (0.49, 2.07)	0.982	208	0.58 (0.39, 0.86)	0.012 **
Pain in last 3 months	228	1.03 (0.65, 1.63)	0.884	219	0.85 (0.63, 1.15)	0.252
**FU2 (6 months)**						
New pain or injury ^2^	115	0.48 (0.13, 1.73)	0.227	112	0.60 (0.24, 1.49)	0.236
Pain in last 3 months	116	0.74 (0.32, 1.69)	0.429	116	0.85 (0.37, 1.99)	0.683

CI = confidence intervals; OR = odds ratio. Results from logistic regression models with cluster robust standard errors to account for individual clustering within worksites (** *p* < 0.05); ^1^ Adjusted model with fixed effects for matched pairs and for age, race, and job title. ^2^ New injury or pain reported by the worker on FU1 /FU2 survey since baseline survey.

**Table 4:** Effects of the ARM intervention on physical activity and dietary behaviors from baseline to FU2 (6 months).

Outcome Variable	Unadjusted			Adjusted ^1^		
	*N*	B (95% CI)	*p-*Value	*N*	B (95% CI)	*p-*Value
Recreational physical activity	97	12.54 (−24.42, 49.51)	0.462	84	35.20 (5.35, 65.04)	0.026 **
Dietary balance	116	0.83 (−0.62, 2.28)	0.229	100	1.05 (−0.02, 2.13)	0.054 *
Healthy diet	118	0.63 (0.33, 1.59)	0.173	101	0.63 (−0.17, 1.43)	0.008 **
Unhealthy diet	116	−0.07 (−1.11, 0.99)	0.89	100	−0.12 (−0.81, 0.56)	0.691

B = regression coefficient; CI = confidence intervals. Results from linear regression models with cluster robust standard errors to account for individual clustering within worksites (* *p* <0.1, ** *p* <0.05). ^1^ Adjusted with fixed effects for matched pairs and age, sex, race, title, and trade.

**Table 5:** Effects of the ARM intervention on Working Conditions and Enterprise Outcomes from baseline to FU1 (1 month) and FU2 (6 months).

Outcome Variable	Unadjusted			Adjusted ^1^		
	*N*	B (95% CI)	*p-*Value	*N*	B (95% CI)	*p-*Value
FU1 (1 month)						
Ergonomic practices	182	0.00 (−0.21, 0.20)	0.953	182	0.20 (0.09, 0.31)	0.002 **
Physically demanding work	208	0.17 (−0.05, 0.37)	0.121	174	0.17 (−0.06, 0.40)	0.129
Work limitations (8-item)	216	0.11 (−0.08, 0.30)	0.225	179	0.09 (−0.06, 0.24)	0.212
**FU2 (6 months)**						
Physically demanding work	114	−0.14 (−0.51, 0.23)	0.407	100	−0.25 (−0.41, −0.08)	0.008 **
Work limitations (8-item)	119	0.02 (−0.08, 0.13)	0.641	102	0.04 (−0.07, 0.15)	0.432

B = regression coefficient; CI = confidence intervals. Results from linear regression models with cluster robust standard errors to account for individual clustering within worksites (** *p* < 0.05). ^1^ Adjusted with fixed effects for matched pairs and age, gender, race, title, and trade.
